# Focal seizures in a patient with myotonic disorder type 2 co-segregating with a *chloride voltage-gated channel 1* gene mutation: a case report

**DOI:** 10.1186/s13256-016-0958-8

**Published:** 2016-06-07

**Authors:** Leema Reddy Peddareddygari, Arman Singh Grewal, Raji Paul Grewal

**Affiliations:** 1 The Neuro-genetics Institute, 501 Elmwood Ave, Sharon Hill, PA USA; 2 Neuroscience Institute, Saint Francis Medical Center, 601 Hamilton Avenue, Trenton, NJ USA

**Keywords:** Myotonic dystrophy type 2, Seizures, Myotonia, *CNBP* and *CLCN1* gene mutations, Case report

## Abstract

**Background:**

Myotonic dystrophy type 2 is a multisystem disorder with both neurological and non-neurological signs and symptoms. Seizures are not a commonly associated neurological feature of this disorder.

**Case presentation:**

A 57-year-old white American man presented with a long history of clinical and electrophysiological features of a myotonic disorder. He also developed multiple episodes of focal seizures and underwent a series of investigations which showed no structural or metabolic etiology. Genetic testing revealed that he had an expansion mutation in *CCHC-type zinc finger, nucleic acid binding protein* gene confirming the diagnosis of myotonic disorder type 2 and carried a mutation in the *chloride voltage-gated channel 1* gene.

**Conclusions:**

We report a rare association between myotonic dystrophy type 2 and a seizure disorder. The pathophysiology of a possible relationship between these two neurological conditions is discussed.

## Background

The myotonic dystrophies are a group of genetic disorders characterized by both neurological and non-neurological manifestations [1]. Myotonic dystrophy type 1 (DM1) is caused by (CTG)n repeat expansions in 3′ untranslated region of the *dystrophia myotonica protein kinase (DMPK)* gene, while myotonic dystrophy type 2 (DM2) is caused by a tetranucleotide repeat (CCTG)n in the first intron of the *CCHC-type zinc finger, nucleic acid binding protein (CNBP)* gene.

Progressive weakness and myotonia are common neurological features in both DM1 and DM2; however, seizures are not typically associated with either of these disorders. We report the case of a patient with genetically confirmed myotonic dystrophy co-segregating with a *chloride voltage-gated channel 1* (*CLCN1*) gene mutation and partial seizures.

## Case presentation

We describe the case of a 57-year-old white American man with a history of weakness who presented for genetic confirmation of a myotonic disorder. He had been evaluated and diagnosed as having a myotonic disorder at age 27 years when he presented for complaints of stiffness. This diagnosis was made after an electromyography (EMG) test was performed. Over the ensuing 30 years, he developed proximal muscle weakness more marked in his legs than arms with no other significant neurological complaints. A neurological examination at age 57 years showed no abnormalities of his mental status; a cranial nerve examination and cerebellar testing showed no abnormalities, and a sensory examination testing proprioception, vibration, light touch, and pin prick sensibility showed no abnormalities. He had normoactive reflexes with flexor plantar responses. An examination of power revealed Medical Research Council (MRC) grade 4/5 weakness in tests of internal and external rotation, and in deltoid, biceps, and hip flexion. In the remaining muscles, he had normal strength. His muscle bulk was normal and although he had no percussion myotonia, there was a delay in releasing a handshake consistent with action-induced myotonia. Tests of gait were normal.

There was no family history of anyone with a similar disorder but his father died at age 56 years from heart disease and there was no other history available on the paternal side. His paternal ancestors came from Romania but originally were of German descent. His mother died at age 94 years from breast cancer and had no clinical features to suggest she was affected by a myotonic disorder.

Multiple recent EMGs had been performed showing the presence of myotonic discharges with no convincing evidence of myopathy. Genetic testing was performed confirming a diagnosis of DM2, an additional mutation was also detected in his *CLCN1* gene (refer to Genetic testing subsection).

He was seen in follow up and several years after the confirmation of the diagnosis, he presented with four episodes of mild weakness of his right arm, leg and face associated with numbness and tingling in the same distribution. He also developed slurring of his speech during these episodes which lasted for approximately 2 minutes following a return to his baseline in 10 minutes. He initially went to our emergency room where a diagnosis of transient ischemic attack was made and investigations were performed including a cerebral magnetic resonance imaging and magnetic resonance angiography, carotid ultrasound, and an echocardiogram. All these tests and routine serum chemistries were normal or negative. These episodes recurred three more times over a course of 6 months. The episodes were stereotypical with no clear initiating event and with full recovery to baseline within 10 minutes. A repeat neurological examination showed no significant change from that initially performed. A routine electroencephalogram (EEG) was done 1 month after the last episode showing slowing in his left cerebral hemisphere with no other abnormality detected. He was diagnosed with focal seizures. Approximately 4 months after the last episode, a repeat routine EEG was done and was normal. This was followed up with a 48-hour EEG recording which was also normal. Over the course of the next 24 months, no further seizure episodes were noted without him taking an anticonvulsant medication.

### Genetic testing

Genetic testing was performed by a commercial company and analysis for expansions in the *DMNK* and *CNBP* genes. However, since he had a prior diagnosis of myotonia congenita, analysis of the skeletal muscle chloride channel, *CLCN1* gene was also performed. The results showed no genetic evidence for DM1; testing for DM2 showed a mutant-expanded intronic allele of greater than 372 CCTG repeats with the wild-type allele of 140 repeats in *CNBP* gene. In addition, a heterozygous mutation in the *CLCN1* was also detected, rs149729531, c.501C>G, p.F167L.

## Discussion

Our patient has genetically confirmed DM2 with an associated *CLCN1* mutation. It is likely that he inherited the mutation from his father who died relatively young and for whom there is little history available. Alternatively, it may represent a spontaneous mutation. Mutations in the *CLCN1* gene can cause either autosomal dominant Thomsen’s type myotonia or recessive Becker’s type. It is interesting that a number of patients with DM2 co-segregating heterozygous recessive mutations in *CLCN1* have been reported [2]. It has been suggested that the patients with DM2 without the associated *CLCN1* mutations are less likely to seek medical attention perhaps indicating that in them, the disease is less phenotypically severe. Recently there have been two case reports of patients with DM who had seizures. The first is that of a patient with the clinical features of DM and epilepsy but without genetic confirmation of diagnosis [3]. In another study, a patient with genetically confirmed DM2 was noted to have a long history of generalized epilepsy [4]. The episodes experienced by our patient are most consistent with focal seizures without impairment of consciousness or awareness [5]. Our patient is the first to be described with partial seizures associated with mutations in both the *CNBP* and *CLCN1* genes. The specific mutation in the *CLCN1* gene in our patient has been previously reported in individuals with Becker’s type or recessive generalized myotonia [6].

There are several links between myotonic disorders and epilepsy. Both are episodic disorders and the treatment of myotonia can include anti-epilepsy medications [7]. The *CNBP* gene is expressed in both peripheral and central nervous system tissues [1]. Until recently, it was thought that the chloride channel protein 1 (ClC-1) protein, the product of the *CLCN1* gene, was not expressed in brain tissue. However, a recent report suggests that *CLCN1* is expressed in the mouse thalamus and cortex [8]. In this study, in addition to this animal data, a patient with epilepsy is presented. A mutation in the *CLCN1* gene is reported and it is hypothesized that this disease-producing mutation is the cause of the seizure disorder in this patient [9]. This supports other research that indicates that there is significant variability in *CLCN1* transcripts in both muscle and brain tissues that could contribute to the phenotypic variability observed in patients with myotonic disorders [8, 10].

In our patient, it is possible that the mutations in *CNBP* or *CLCN1* genes alone or in combination are involved in the pathophysiology of the seizure.

## Conclusions

This report describes the rare case of a patient with mutations in *CNBP* and *CLCN1* genes presenting with DM2 and a seizure disorder. This co-occurrence of these two episodic disorders could indicate a shared pathophysiology.

## Abbreviations

ClC-1, chloride channel protein 1; *CLCN1*, *chloride voltage-gated channel 1* gene; *CNBP*, *CCHC-type zinc finger, nucleic acid binding protein* gene; DM1, myotonic dystrophy type 1; DM2, myotonic dystrophy type 2; *DMPK*, *dystrophia myotonica protein kinase*; EEG, electroencephalogram; EMG, electromyography; MRC, Medical Research Council
